# Incidence Rates and Case-Fatality Rates of Cerebral Vein Thrombosis: A Population-Based Study

**DOI:** 10.1161/STROKEAHA.121.034202

**Published:** 2021-08-10

**Authors:** Emanuele Rezoagli, Aldo Bonaventura, Jonathan M. Coutinho, Alessandra Vecchié, Vera Gessi, Roberta Re, Alessandro Squizzato, Fulvio Pomero, Matteo Bonzini, Walter Ageno, Francesco Dentali

**Affiliations:** Department of Medicine and Surgery, University of Milano-Bicocca, Monza, Italy (E.R.).; Department of Emergency and Intensive Care, San Gerardo Hospital, Monza, Italy (E.R.).; Department of Internal Medicine, ASST Sette Laghi, Varese, Italy (A.B., A.V., V.G.).; First Clinic of Internal Medicine, Department of Internal Medicine, University of Genoa, Italy (A.B.).; Department of Neurology, Amsterdam University Medical Centers, University of Amsterdam, the Netherlands (J.M.C.).; Presidio Ospedaliero S. Andrea, ASL VC, Vercelli, Italy (R.R.).; Medicine and Surgery (A.S.), University of Insubria, Varese/Como, Italy.; Department of Medicine and Surgery (W.A., F.D.), University of Insubria, Varese/Como, Italy.; Internal Medicine, Michele e Pietro Ferrero, Verduno, Cuneo, Italy (F.P.).; Department of Clinical Sciences and Community Health, University of Milan, IRCCS Policlinico Fundation, Italy (M.B.).

**Keywords:** cerebrovascular disorders, cerebral hemorrhage, epidemiology, incidence, intracranial thrombosis, mortality, venous thrombosis

## Abstract

Cerebral vein thrombosis (CVT) incidence is estimated to be >10 per 1 000 000 per year. Few population-based studies investigating case-fatality rates (CFRs) and pyogenic/nonpyogenic CVT incidence are available. We assessed trends in CVT incidence between 2002 and 2012, as well as adjusted in-hospital CFRs and incidence of hospital admissions for pyogenic/nonpyogenic CVT in a large Northwestern Italian epidemiological study.

**Methods:**

Primary and secondary discharge diagnoses of pyogenic/nonpyogenic CVT were identified using International Classification of Diseases, Ninth Revision, Clinical Modification codes 325, 671.5, and 437.6. Age, sex, vital status at discharge, length of hospital stay, and up to 5 secondary discharge diagnoses were collected. Concomitant presence of intracerebral hemorrhage (ICH) was registered, and comorbidities were assessed through the Charlson comorbidity index.

**Results:**

A total of 1718 patients were hospitalized for CVT (1147 females—66.8%; 810 pyogenic and 908 nonpyogenic CVT, 47.1% and 52.9%, respectively), with 134 patients (7.8%) experiencing a concomitant ICH. The overall incidence rate for CVT was 11.6 per 1 000 000 inhabitants with a sex-specific rate of 15.1 and 7.8 per 1 000 000 in females and males, respectively. CVT incidence significantly increased in women during time of observation (*P*=0.007), with the highest incidence being at 40 to 44 years (27.0 cases per 1 000 000). In-hospital CFR was 3%, with no difference between pyogenic/nonpyogenic CVT. Patients with concomitant ICH had a higher in-hospital CFR compared with patients without ICH (7.5% versus 2.7%; odds ratio, 2.96 [95% CI, 1.45–6.04]). In-hospital CFR progressively increased with increasing Charlson comorbidity index (*P*=0.003). Age (odds ratio, 1.03 [95% CI, 1.02–1.05]), Charlson comorbidity index ≥4 (odds ratio, 4.33 [95% CI, 1.29–14.52]), and ICH (odds ratio, 3.05 [95% CI, 1.40–6.62]) were independent predictors of in-hospital mortality.

**Conclusions:**

In a large epidemiological study, CVT incidence was found to be comparable to the one registered in population-based studies reported after the year 2000. CVT incidence increased among women over time. In-hospital CFR was low, but not negligible, in patients with concomitant ICH. Age, ICH, and a high number of comorbidities were independent predictors of in-hospital mortality. Pyogenic CVT was not a predictor of in-hospital CFR, although its high proportion was not confirmed by internal validation.

## Abstract 2

Supplemental Digital Content is available in the text.

Cerebral vein thrombosis (CVT) is a cerebrovascular disease that may occur at any age and represents 0.5% of all strokes.^1,2^ First reports on CVT, extrapolated from autopsy series, suggested a CVT incidence of 1 to 2 cases per 1 000 000 people.^3^ Other studies reported higher incidences, ranging from 2 to 5 cases per 1 000 000 per year.^1,4,5^ A recent population-based study in the Netherlands observed an overall incidence of 13.2 per 1 000 000 per year, with a higher proportion observed in women.^2^ In most cases, pathological conditions leading to thrombosis in the venous circulation of the brain are associated with a nonpyogenic CVT, whereas pyogenic CVT may complicate head traumas and result from the local extension of an infection from the cranial sinuses.^5,6^

In recent years, the widespread use of noninvasive neuroimaging has allowed early diagnosis and detection of less clinically severe cases of CVT, potentially explaining an increase in CVT incidence.

Few controversial data are available regarding in-hospital mortality for CVT. Despite CVT being previously associated with poor prognosis,^7,8^ recent studies have been reporting more favorable outcomes, with mortality after the index event being ≈4% at discharge.^9^

We thus designed a large epidemiological Italian study with the aim of evaluating CVT incidence, in-hospital mortality rate, and factors associated with mortality.

## Methods

This article adheres to the American Heart Association Journals’ implementation of the Transparency and Openness Promotion Guidelines (available online at http://www.ahajournals.org/TOP-guidelines). The data that support the findings of this study are available from the corresponding author upon reasonable request.

### Patient Selection and Eligibility

The study was conducted in adherence to the Declaration of Helsinki. Hospital ethics committee of the coordinating center (University Teaching Hospital – Ospedale di Circolo e Fondazione Macchi, Varese, Italy) approved the study and waived the need of written informed consent due to the retrospective nature of the study.

CVT admissions were systematically searched in the hospital discharge regional databases of the regional Center for Health Statistics of Lombardy and Piedmont, in Northwestern Italy (14 820 000 inhabitants). The time of the analysis included CVT admissions between January 1, 2000, and December 31, 2012. Patients with pyogenic and nonpyogenic CVT were considered and identified using the *International Classification of Diseases, Ninth Revision, Clinical Modification* codes (*ICD-9-CM*, codes: 325, 671.5, and 437.6). Hospitalized patients could be evaluated according to data collected in the regional databases of Lombardy and Piedmont. Only patients with a first episode of CVT were eligible.

The following variables were available for all patients: sex, date of birth, marital status, resident and hospital ZIP codes, date of hospital admission and discharge, hospital department of admission and discharge, diagnosis, length of hospital stay (in days), health status at discharge, one primary and up to 5 secondary discharge diagnoses, and one primary and up to 4 in-hospital diagnostic procedures codes. Any patient identifier was not reported in the database, in compliance with the national privacy law and guidelines for Good Clinical Practice (revised version 2016).

To allow for the identification of multiple hospital admissions, every patient had an encoded identification number. Only the first hospital admission with a CVT diagnosis during the study period has been included in the database so as to exclude recurrences. Patients admitted to a hospital and then transferred to another hospital were counted as experiencing a single event.

The date of hospitalization is considered as the day of admission to the first hospital, while the diagnosis was defined by the discharging hospital. Since we did not have information before the year 2000, we excluded all events occurred during 2000 and 2001. Patients hospitalized with a CVT diagnosis and a previous hospital admission with the same diagnosis in 2000 and 2001 were not considered as incident cases and were excluded from the analysis.

### Study End Points

The primary end point of this study was to estimate the incidence of CVT and in-hospital mortality rate during the period of observation. Secondary end points included: (1) stratification of in-hospital mortality rate by sex; (2) stratification of in-hospital mortality rate by pyogenic versus nonpyogenic CVT; and (3) evaluation of predictors of in-hospital mortality among hospitalized patients with CVT.

### Definition of Comorbidities

The burden of comorbidities for each patient was estimated in through the Charlson comorbidity index (CCI), a 17-item weighted score accounting for the number and the severity of comorbid diseases.^10^ The score was not adjusted for age.

### Statistical Analysis

The distribution of continuous data was examined through the Kolmogorov-Smirnov test. Normal distribution is presented as mean±SD, whereas non-normal distribution is given as median and interquartile range.

Difference among continuous variables was tested using an unpaired Student *t* test or Mann-Whitney *U* test, as appropriate.

Annual incidence rates were calculated as the number of cases occurring in each study year over the total number of inhabitants of Lombardy and Piedmont (Italy). Sex-specific incidence rates of CVT were also calculated. Age was stratified in 5-year age categories dividing incidence cases of CVT by the population of the corresponding 5-year time period according to the databases of the Italian National Institute of Statistics.

Incidence rate time trends across the study period (2002–2012) were compared by sex using linear regression models and confirmed through log-transformed age-standardized rates. Each annual rate was adjusted for the overall number of incident cases.

In-hospital case-fatality rate (CFR) was calculated as the proportion of fatal cases during hospitalization over the total incident cases. Potential predictors of mortality were chosen among those considered clinically relevant and evaluated through a univariate logistic regression model: age, sex, CCI, intracerebral hemorrhage (ICH), and pyogenic versus nonpyogenic CVT. To avoid an underpowered analysis, all patients with a CCI≥4 were grouped together. A backward stepwise regression was used to evaluate independent risk factors for in-hospital mortality. Relative risk (RR) and the corresponding 95% CI are presented for all variables.

Difference between each single item of CCI stratified by in-hospital mortality was assessed by the Fisher exact test.

For all statistical analyses, a 2-sided *P*<0.05 was considered statistically significant. Statistical analyses were performed using STATA-12 (Stata Corp LP, College Station, Austin, TX), IBM SPSS Statistics for Mac, Version 26.0 (IBM CO, Armonk, NY), and GraphPad Prism, version 8.2 for Windows (GraphPad Software, La Jolla, CA; www.graphpad.com).

The study was conducted and reported based on the Reporting of Studies Conducted Using Observational Routinely Collected Data guidelines for observational studies.^11^ A completed checklist and flow diagram was reported in the Data Supplement.

## Results

### General Characteristics of the Cohort

During the time of observation (2002–2012), 1718 patients with CVT (511 in Piedmont and 1207 in Lombardy) were hospitalized. Pyogenic CVT was 810 (47.1%). Two out of 3 patients of the study population were female (n=1147). Baseline patient characteristics are summarized in Table 1. Women were younger than men and men presented a higher burden of comorbidities (Table I in the Data Supplement).

**Table 1.**
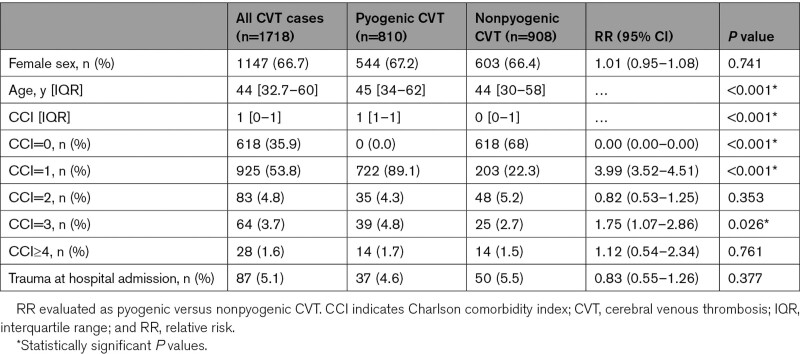
Baseline Characteristics of the Study Population

**Figure 1.**
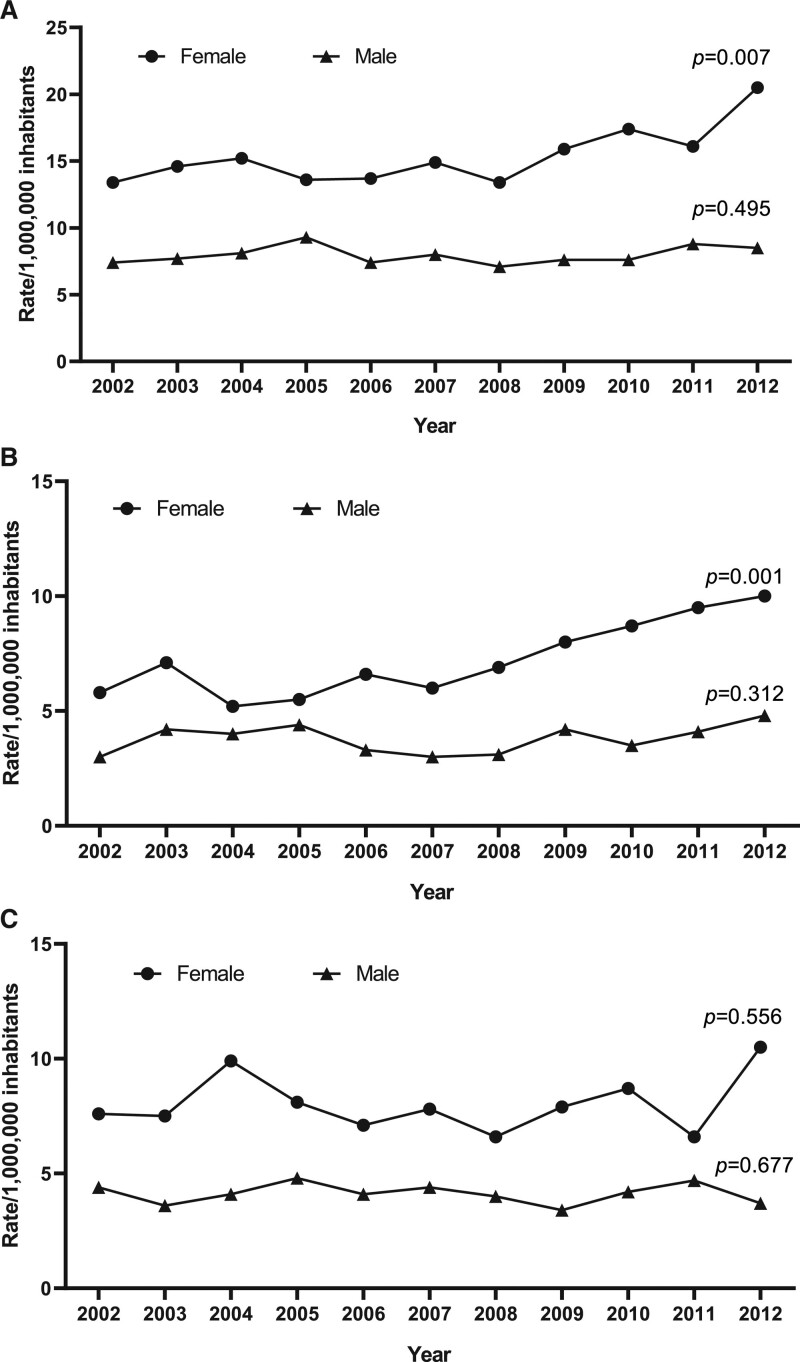
**Incidence rates of cerebral vein thrombosis (CVT) across the period of observation.** **A**, A statistically significant, progressive increase in CVT was observed in female individuals from 2002 to 2012 (*P*=0.007), while incidence rates remained stable among male subjects. **B**, A progressive increase in pyogenic CVT incidence over the time of observation was found in women (*P*=0.001) but not in men. **C**, Nonpyogenic CVT incidence did not show any statistically significant change over time neither in male nor in female subjects.

The overall incidence of CVT across the study period was 11.6 per 1 000 000 inhabitants. Sex-specific incidence was 7.8 per 1 000 000 inhabitants in males and 15.1 per 1 000 000 in females (*P*<0.001). CVT incidence rate increased in females during the observation period (from 13.4 in 2002 to 20.5 per 1 000 000 inhabitants in 2012, *P*=0.007; Figure 1A) mainly driven by an increased incidence in pyogenic CVT (*P*=0.001; Figure 1B). The incidence of nonpyogenic CVT in both sexes did not significantly differ over time (Figure 1C).

The overall incidence of pyogenic and nonpyogenic CVT across the study period was 5.5 and 6.1 per 1 000 000 inhabitants, respectively. Sex-specific incidence in pyogenic CVT was 3.7 per 1 000 000 inhabitants in males and 7.1 per 1 000 000 in females (*P*<0.001). Sex-specific incidence in nonpyogenic CVT was 4.1 per 1 000 000 inhabitants in males and 7.9 per 1 000 000 in females (*P*<0.001).

**Figure 2.**
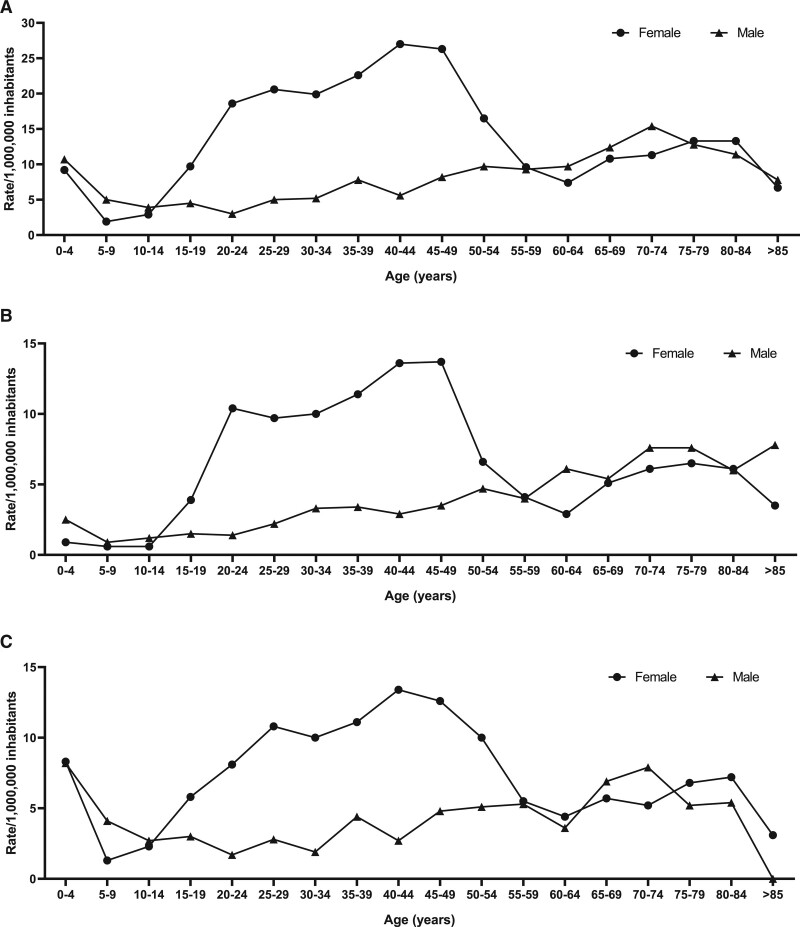
**Cerebral vein thrombosis (CVT) incidence across age groups.** **A** shows a net, higher incidence of CVT among women until the age of 40–49, with a progressive and slight increase observed among men until older age. **B** shows that the incidence of pyogenic CVT increases until the age of 40–49 in female individuals and then declines, while it remains quite stable among male subjects. **C** shows a stable higher incidence of nonpyogenic CVT in women from 20 to 54 years, while the incidence rate is stable in men across all age groups.

Among women, CVT incidence was higher in those aged 40 to 44 years (27.0 cases per 1 000 000 inhabitants), whereas among men the highest incidence was found at 70 to 74 years (15.4 per 1 000 000 inhabitants; Figure 2A). Incidence rates of pyogenic and nonpyogenic CVT were higher in females aged 20 to 50, while it was similar in both sexes before and after that range of age (Figure 2B and 2C).

### Clinical Outcomes

**Figure 3.**
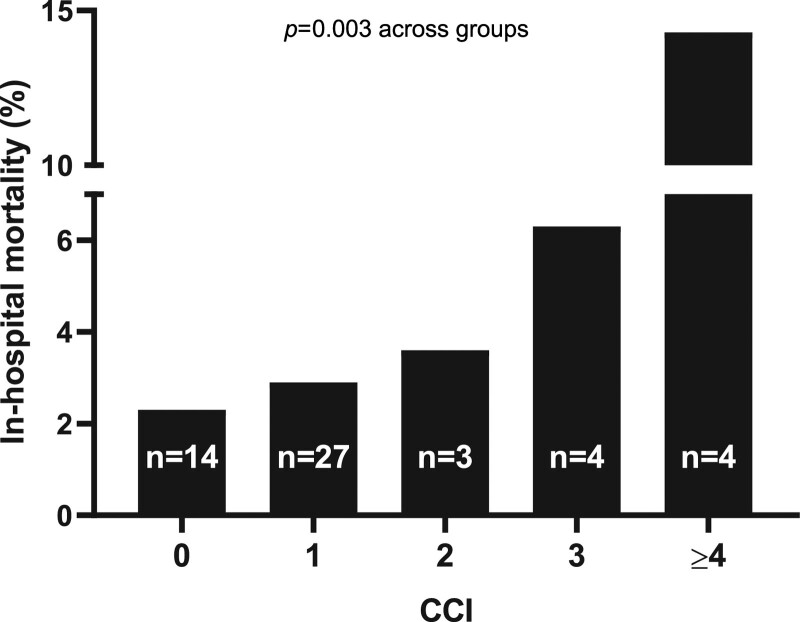
**In-hospital mortality rate according to Charlson comorbidity index (CCI).** A progressive increase in the percentage of dead patients was observed with increasing CCI score (*P* for χ^2^ test=0.003).

The median length of hospitalization was 12 (8–18) days, slightly higher among patients with pyogenic versus nonpyogenic CVT (*P*=0.002, Table 2). During hospitalization, 52 patients (3%) died, but no statistically significant difference was found neither between pyogenic and nonpyogenic CVT nor between males and females (Table 2 and Table I in the Data Supplement, respectively). A higher in-hospital mortality was observed with increasing CCI scores (*P*=0.003, Figure 3).

**Table 2.**
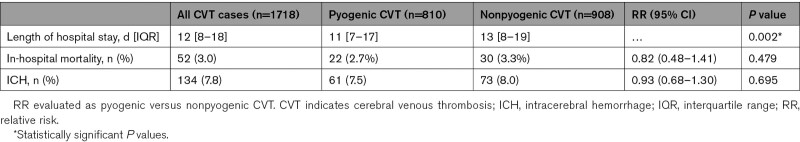
Outcomes of the Study Population

Concomitant ICH was detected in 134 patients (7.8%). Patients with ICH experienced higher mortality compared with those without it (7.5% [n=10] versus 2.7% [n=42]; RR, 2.96 [95% CI, 1.45–6.04], *P*=0.003). No difference in the presence of ICH was observed neither between pyogenic and nonpyogenic CVT nor between males and females (Table 2 and Table I in the Data Supplement, respectively).

Using a multivariate logistic regression analysis, age (RR, 1.03 [95% CI, 1.02–1.05], *P*<0.001), CCI≥4 (RR, 3.62 [95% CI, 1.25–10.50], *P*=0.018), and ICH (RR, 2.81 [95% CI, 1.38–5.73], *P*=0.005) were positive independent predictors of in-hospital mortality (Table 3).

**Table 3.**
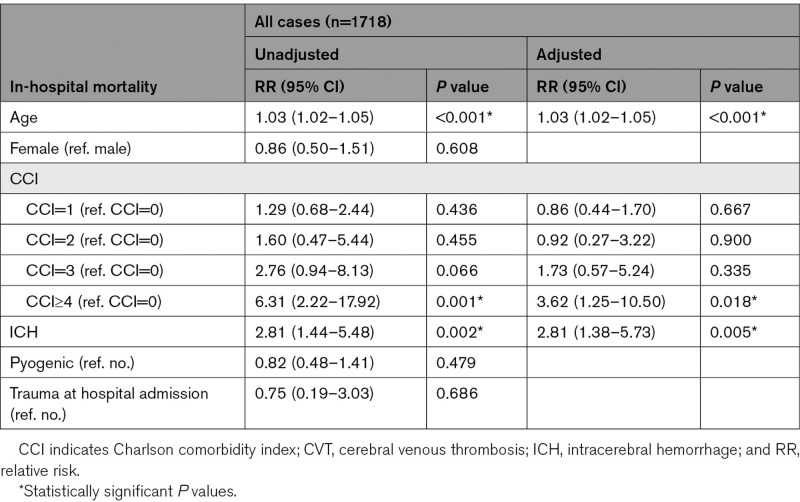
Logistic Regression Analysis Evaluating Predictors of In-Hospital Mortality in Patients With CVT at Diagnosis

A comprehensive description on the association among the occurrence of each item of the CCI with in-hospital mortality was reported in Table II in the Data Supplement.

## Discussion

The main findings of this study, based on a large database from Northwestern Italy, are (1) a higher incidence of CVT compared with the one previously reported in the literature before 2000 and comparable findings to population-based studies reported after 2000, (2) a more common occurrence of CVT in middle-aged women (40–49 years) and in older men (70–74 years), (3) an increased overall incidence across the time of observation (2000–2012) in women, and (4) a higher rate of in-hospital mortality among patients with concomitant ICH.

First reports derived from autopsy series dating back to few decades ago showed a CVT incidence ranging from 1 to 5 cases per 1 000 000 per year.^1,3,5^ Later on, other clinical reports from different areas of the world described an increasing incidence, ranging from 12.3 per 1 000 000 per year (ie, 2001–2004)^12^ to up to 15.7 per 1 000 000 per year (ie, 2005–2011).^13^ Results from a population-based study performed in the Netherlands reported higher rates (13.2 cases per 1 000 000 per year between 2008 and 2010), with striking sex-related differences.^2^ Incidence rates were 18.6 per 1 000 000 per year in women and 7.5 per 1 000 000 per year in men.^2^

A report from Finland recently confirmed data reported in a study conducted in the Netherlands: an overall incidence rate of 13.3 per 1 000 000 per year (ie, 2005–2014) and also a different sex-related incidence of CVT. Such sex-related difference showed a higher CVT incidence in women younger than 55 years as opposed to a lower incidence in those older than 55 years, compared with man of the same range of age that showed the exact opposite trend.^14^ In our study, the incidence rate was in line with the one reported by Coutinho et al^2^ and higher than previously reported, with a net prevalence of women. In addition, in our cohort, the increased incidence of CVT in women has been a consistent finding throughout the 10-year observation period, while the same has not been found in men. An explanation for this may be the progressive improvement of diagnostic techniques allowing for a prompt recognition of those cases presenting with early and minimal symptoms since CVT might present with a wide spectrum of symptoms.^15^ Another possible explanation might be the presence of sex-specific risk factors, such as the use of oral contraceptives^3^ and the higher proportion of milder symptoms, such as headache, at CVT onset in women.^16^

The highest incidence rates observed in our study occurred between 40 and 49 years in women and between 70 and 74 years in men. This is in line with recent data from patients with a first CVT episode showing that women were younger than men (39±15 versus 42±19 in Porceddu et al^17^; 34 [25–47] versus 42 [33–57] in Coutinho et al^16^). The younger age of CVT incidence in women may be well explained by some known sex-based risk factors, such as pregnancy, puerperium, hormone replacement therapy, and oral contraceptives.^16,17^ In contrast, the incidence in older men might be explained by a higher risk of dehydration in such a selected population.^5^

An interesting finding from our database is the high number of pyogenic CVT (almost 50%). Infections involving central nervous system or the head (including ear, sinus, mouth, face, and neck) were recognized as a risk factors for CVT in 10% of patients diagnosed with CVT.^9^ Ferro et al^9^ reported that 12.3% of patients presented with an underlying infection as risk factor for CVT and this number was in line with a previous study.^18^ Also in developing countries, the percentage of pyogenic CVT is not >20% of CVT cases.^19^ Borhani Haghighi et al,^20^ however, reported >80% of pyogenic CVT in their study. This finding may be due to coding errors leading to an overdiagnosis of pyogenic CVT or alternatively to an underreporting of nonpyogenic CVT.^21^ In light of all these findings, our data about pyogenic CVT may be explained by an increased ability in diagnosing this entity thanks to advancements in imaging techniques and to having acquired more data on this topic over the years.^22^ However, the high incidence of pyogenic CVT reported in our data should be considered with some caution as the diagnosis derived from *ICD* classification. For this reason, we cannot exclude an overrepresentation of this entity since a thorough chart review was not possible with the available data.

CVT has a generally favorable outcome, with a mortality rate ranging from 1% to 4% for in-hospital mortality and 8% to 10% during long-term follow-up.^2,9,17,20,23–25^ Most patients with a first CVT event experience a complete or partial recanalization that was associated with a good neurological outcome.^26^ However, CFR can be higher in the acute phase (around 4%) usually because of transtentorial herniation following a large hemorrhage, diffuse brain edema, or multiple parenchymal lesions.^23,25^ In our cohort, we observed a CFR of 3% that confirms previously published data in European and extra-European countries.^9,20,25,27^ In addition, patients with ICH experienced a nearly 3-fold increased risk of in-hospital mortality compared with those without ICH at the time of presentation. Although we do not have data about recovery from the acute event, ICH was described to potentially worsen the prognosis of patients with CVT, as reported either from European data by Dentali et al,^25^ or in a large multicenter study from the Middle-East.^28^ Indeed, we found that ICH was a strong predictor of in-hospital mortality along with age and CCI≥4, thus confirming previous data.^20^

Strengths and limitations must be acknowledged in our work. The strength of this study relies on the large number of patients included in the analysis that has been performed across a time-lapse of 10 years, thus allowing for a sufficiently accurate evaluation of incidence, CFR, and mortality predictors of CVT. Limitations include the use of *ICD-9-CM* diagnosis codes that could not be validated by an internal cohort. This may have led to a poor sensitivity and specificity in capturing CVT, especially the pyogenic form. To reduce false-positive diagnoses, we only included patients with first and second codes related to CVT. Although CCI may yield a low sensitivity for younger patients, like those with CVT, this was not the case in our study as half of the patients aged 40 or less (52.6%, n=380) presented a history of a cerebrovascular accident with minor or no sequelae as well as transient ischemic attacks, thus largely fulfilling this point in the CCI score calculation. Due to the observational design of the study and the unavailability of data on treatments, thorough information on the clinical history of CVT—including in and out of hospital events—may have been missed. Furthermore, we were not able to evaluate the impact of antithrombotic drugs on outcomes.

As for the trauma origin of CVT, we could solely describe the presence of a traumatic event at hospital admission but we could not be selective in terms of origin site (ie, head and neck). This might explain the higher proportion of trauma in our current findings compared with other data reported from our group in the CEVETIS study.^25^

Finally, although the data we presented are based on a large sample size and include age, sex, comorbidities, and presence or absence of pyogenic CVT, they caught events occurred until 2012, thus they might not exactly reflect the current clinical picture of CVT. However, until very recently, therapeutic strategies have not changed over the last years, with the large majority of patients epidemiological study show a higher incidence of CVT compared with previous findings, that progressively increased during the time receiving heparin (unfractionated or low molecular weight) followed by warfarin.

## CONCLUSIONS

In conclusion, results deriving from this large of observation, especially in women. Prognosis was confirmed to be generally good, with low mortality, but ICH was found to greatly increase the CFR when present at the time of diagnosis. In addition, ICH, age, and CCI≥4 were found to independently predict in-hospital mortality. Finally, we reported a higher number of pyogenic versus nonpyogenic CVT, although this finding needs to be carefully considered due to the observational design of the study and to the *ICD* based diagnosis of CVT. Future prospective studies with long follow-up are warranted to investigate post-CVT disability, long-term mortality, and management of antithrombotic therapy. With regard to the latter, it is worth investigating whether direct oral anticoagulants may change the natural history of CVT since they have been poorly studied until now in this setting.^29^

## Sources of Funding

None.

## Disclosures

Drs Bonaventura and Vecchié received a travel grant from Kiniksa Pharmaceuticals Ltd to attend the 2019 American Heart Association (AHA) Scientific Sessions and receive honoraria from Effetti s.r.l. (Italy) to collaborate on the medical website www.inflammology.org outside the present work. Dr Coutinho reports grants from Boehringer Ingelheim and grants from Bayer outside the submitted work. Dr Ageno has received research support from Bayer and grants for participation in advisory boards from Bayer, Janssen, Portola, Aspen, and Sanofi. The other authors report no conflicts.

## Supplemental Materials

RECORD checklist

RECORD flow diagram

Online Tables I–II

## Supplementary Material


