# Supplementary material for: Incidence Rates and Case-Fatality Rates of Cerebral Vein Thrombosis: A Population-Based Study
Source: Stroke. 2021 Aug 10;52(11):3578–85. doi: 10.1161/STROKEAHA.121.034202 (PMC10082066; doi:10.1161/STROKEAHA.121.034202)
Supplement: Supplementary file 1 [file str-52-3578-s001.pdf]

SUPPLEMENTAL MATERIAL

REporting of studies **C**onducted using **O**bservational **R**outinely-collected **D**ata (**RECORD**) **flow diagram**

Inclusion criteria:
- Hospitalized patients with a first episode of pyogenic or non-pyogenic CVT identified using International Classification of Diseases, 9th Revision, Clinical Modification codes 325, 671.5, and 437.6;
- Data collection from regional databases of the regional Center for Health Statistics of Lombardy and Piedmont, in Northwestern Italy (14,820,000 inhabitants);
- Time frame January 1st, 2000 and December 31st, 2012.

Region – Lombardy
CVT cases, n=1207 – Non-survivors, n=27

Year 2002 - CVT cases 89 - Non-survivors 4
Year 2003 - CVT cases 96 - Non-survivors 2
Year 2004 - CVT cases 104 - Non-survivors 3
Year 2005 - CVT cases 106 - Non-survivors 6
Year 2006 - CVT cases 100 - Non-survivors 0
Year 2007 - CVT cases 105 - Non-survivors 3
Year 2008 - CVT cases 104 - Non-survivors 3
Year 2009 - CVT cases 132 - Non-survivors 1
Year 2010 - CVT cases 129 - Non-survivors 2
Year 2011 - CVT cases 134 - Non-survivors 2
Year 2012 - CVT cases 108 - Non-survivors 1

Region – Piedmont
CVT cases, n=511 – Non-survivors, n=25

Year 2002 - CVT cases 50 - Non-survivors 3
Year 2003 - CVT cases 55 - Non-survivors 2
Year 2004 - CVT cases 52 - Non-survivors 4
Year 2005 - CVT cases 50 - Non-survivors 3
Year 2006 - CVT cases 43 - Non-survivors 2
Year 2007 - CVT cases 46 - Non-survivors 3
Year 2008 - CVT cases 42 - Non-survivors 0
Year 2009 - CVT cases 37 - Non-survivors 3
Year 2010 - CVT cases 49 - Non-survivors 2
Year 2011 - CVT cases 49 - Non-survivors 3
Year 2012 - CVT cases 38 - Non-survivors 0

Overall patient population (Year 2002-2012)
CVT cases, n=1718 – Non-survivors, n=52

Supplementary Table I. Baseline characteristics and outcomes of the study population

	**All CVT cases (n=1,718)**	**Males (n=571)**	**Females (n=1,147)**	**RR (95% CI)**	***p*-value**
**Age, years [IQR]**	44 [32.7-60]	51 [34-66]	42 [32-54]	—	**<0.001**
**CCI [IQR]**	1 [0-1]	1 [0-1]	1 [0-1]	—	**0.003**
**CCI=0, n (%)**	618 (35.9)	198 (34.7)	420 (36.6)	1.06 (0.92-1.21)	0.432
**CCI=1, n (%)**	925 (53.8)	295 (51.7)	630 (54.9)	1.06 (0.97-1.17)	0.207
**CCI=2, n (%)**	83 (4.8)	40 (7.0)	43 (3.7)	0.54 (0.35-0.81)	**0.003**
**CCI=3, n (%)**	64 (3.7)	21 (3.7)	43 (3.7)	1.02 (0.61-1.70)	0.942
**CCI ≥4, n (%)**	28 (1.6)	17 (3.0)	11 (1.0)	0.32 (0.15-0.68)	**0.003**
**Trauma at hospital admission, n (%)**	87 (5.1)	27 (4.7)	60 (5.2)	1.11 (0.71-1.72)	0.655
**ICH, n (%)**	134 (7.8)	50 (8.8)	84 (7.3)	0.84 (0.60-1.17)	0.296
**In-hospital mortality**	52 (3.0)	19 (3.3)	33 (2.9)	0.86 (0.50-1.51)	0.608
**Length of hospital stay, days [IQR]**	12 [8-18]	11 [7-18]	12 [8-18]	—	0.386

Statistically significant *p*-values are in bold character. RR evaluated as females versus males.

Legend. CCI: Charlson comorbidity index. CVT: cerebral venous thrombosis. ICH: intracerebral hemorrhage. IQR: interquartile range. RR: relative risk.

Supplementary Table II. Difference in the proportion of each item of the Charlson Comorbidity Index among CVT patients stratified by in-hospital mortality.

**Charlson Comorbidity Score Item**	**Survivors (n=1666)**	**Non-survivors (n=52)**	**p-value**
Myocardial infarction	7 (0.4)	2 (3.9)	**0.028**
Congestive heart failure	1 (0.1)	2 (3.9)	**0.003**
Peripheral vascular disease	3 (0.2)	0 (0.0)	1.000
Cerebrovascular disease	988 (59.3)	31 (59.6)	1.000
Dementia	4 (0.2)	0 (0)	1.000
Chronic pulmonary disease	9 (0.5)	0 (0)	1.000
Rheumatologic disease	8 (0.5)	0 (0)	1.000
Peptic ulcer disease	2 (0.1)	0 (0)	1.000
Mild liver disease	2 (0.1)	0 (0)	1.000
Diabetes	39 (2.3)	0 (0)	0.629
Diabetes with chronic complications	6 (0.4)	1 (1.9)	0.194
Hemiplegia or paraplegia	20 (1.2)	0 (0)	1.000
Renal disease	11 (0.7)	1 (1.9)	0.309
Any malignancy, including leukemia and lymphoma	67 (4.0)	6 (11.5)	**0.021**
Moderate or severe liver disase	1 (0.1)	0 (0)	1.000
Metastatic solid tumor	20 (1.2)	3 (5.8)	**0.030**
AIDS	5 (0.3)	1 (1.9)	0.169

Statistically significant *p*-values are in bold character.

The RECORD statement – checklist of items, extended from the STROBE statement, that should be reported in observational studies using routinely collected health data.

	Item No.	STROBE items	Location in manuscript where items are reported	RECORD items	Location in manuscript where items are reported
**Title and abstract**					
	1	(a) Indicate the study's design with a commonly used term in the title or the abstract (b) Provide in the abstract an informative and balanced summary of what was done and what was found	Study type - Population based-study/ epidemiological study - reported in the title and in the abstract.	RECORD 1.1: The type of data used should be specified in the title or abstract. When possible, the name of the databases used should be included.<br><br>RECORD 1.2: If applicable, the geographic region and timeframe within which the study took place should be reported in the title or abstract.<br><br>RECORD 1.3: If linkage between databases was conducted for the study, this should be clearly stated in the title or abstract.	Abstract: Background and methods<br><br>Abstract: Background.<br><br>Not applicable
**Introduction**					
Background rationale	2	Explain the scientific background and rationale for the investigation being reported	Abstract and introduction		
Objectives	3	State specific objectives, including any prespecified hypotheses	Introduction and section "study endpoints" of materials and methods		
**Methods**					
Study Design	4	Present key elements of study design early in the paper	Methods:Patient selection and eligilibity		
Setting	5	Describe the setting, locations, and relevant dates, including periods of recruitment, exposure, follow-up, and data collection	Methods:Patient selection and eligilibity		

Participants	6	*(a) Cohort study* - Give the eligibility criteria, and the sources and methods of selection of participants. Describe methods of follow-up<br>*Case-control study* - Give the eligibility criteria, and the sources and methods of case ascertainment and control selection. Give the rationale for the choice of cases and controls<br>*Cross-sectional study* - Give the eligibility criteria, and the sources and methods of selection of participants<br><br>*(b) Cohort study* - For matched studies, give matching criteria and number of exposed and unexposed<br>*Case-control study* - For matched studies, give matching criteria and the number of controls per case	Methods:Patient selection and eligibility<br><br>Not applicable	RECORD 6.1: The methods of study population selection (such as codes or algorithms used to identify subjects) should be listed in detail. If this is not possible, an explanation should be provided.<br><br>RECORD 6.2: Any validation studies of the codes or algorithms used to select the population should be referenced. If validation was conducted for this study and not published elsewhere, detailed methods and results should be provided.<br><br>RECORD 6.3: If the study involved linkage of databases, consider use of a flow diagram or other graphical display to demonstrate the data linkage process, including the number of individuals with linked data at each stage.	Methods:Patient selection and eligibility<br><br>Not applicable<br><br>Not applicable
Variables	7	Clearly define all outcomes, exposures, predictors, potential confounders, and effect modifiers. Give diagnostic criteria, if applicable.	Methods:<br>Patient selection and eligibility.<br>Study endpoints.<br>Definition of comorbidities.	RECORD 7.1: A complete list of codes and algorithms used to classify exposures, outcomes, confounders, and effect modifiers should be provided. If these cannot be reported, an explanation should be provided.	Methods:<br>Patient selection and eligibity.<br>Study endpoints.<br>Definition of comorbidities.
Data sources/ measurement	8	For each variable of interest, give sources of data and details of methods of assessment (measurement).<br>Describe comparability of assessment methods if there is more than one group	Methods:<br>Patient selection and eligibity.<br>Definition of comorbidities.		

Bias	9	Describe any efforts to address potential sources of bias	Methods: Statistical analysis		
Study size	10	Explain how the study size was arrived at	Methods: Statistical analysis		
Quantitative variables	11	Explain how quantitative variables were handled in the analyses. If applicable, describe which groupings were chosen, and why	Methods: Statistical analysis		
Statistical methods	12	(a) Describe all statistical methods, including those used to control for confounding<br>(b) Describe any methods used to examine subgroups and interactions<br>(c) Explain how missing data were addressed<br>(d) *Cohort study* - If applicable, explain how loss to follow-up was addressed<br>*Case-control study* - If applicable, explain how matching of cases and controls was addressed<br>*Cross-sectional study* - If applicable, describe analytical methods taking account of sampling strategy<br>(e) Describe any sensitivity analyses	Methods:<br>Statistical analysis		
Data access and cleaning methods		..	Methods:<br>adherence to TOP guidelines.<br>Patient selection and eligibility.<br>Statistical analysis.	RECORD 12.1: Authors should describe the extent to which the investigators had access to the database population used to create the study population.	Methods:<br>adherence to TOP guidelines.<br>Patient selection and eligibility.<br>Statistical analysis.

				RECORD 12.2: Authors should provide information on the data cleaning methods used in the study.	Methods: Patient selection and eligibility. Statistical analysis.
Linkage		..	Not applcable	RECORD 12.3: State whether the study included person-level, institutional-level, or other data linkage across two or more databases. The methods of linkage and methods of linkage quality evaluation should be provided.	Not applicable
**Results**					
Participants	13	(a) Report the numbers of individuals at each stage of the study (*e.g.*, numbers potentially eligible, examined for eligibility, confirmed eligible, included in the study, completing follow-up, and analysed)<br>(b) Give reasons for non-participation at each stage.<br>(c) Consider use of a flow diagram	Results: general characteristics of the cohort.<br>We provided a flow diagram.	RECORD 13.1: Describe in detail the selection of the persons included in the study (*i.e.*, study population selection) including filtering based on data quality, data availability and linkage. The selection of included persons can be described in the text and/or by means of the study flow diagram.	Methods: Patient selection and eligibility.<br>Results: general characteristics of the cohort.<br>We provided a flow diagram.
Descriptive data	14	(a) Give characteristics of study participants (*e.g.*, demographic, clinical, social) and information on exposures and potential confounders<br>(b) Indicate the number of participants with missing data for each variable of interest<br>(c) *Cohort study* - summarise follow-up time (*e.g.*, average and total amount)	Results: general characteristics of the cohort.		
Outcome data	15	*Cohort study* - Report numbers of outcome events or summary measures over time<br>*Case-control study* - Report numbers in each exposure	Results: clinical outcomes.		

		category, or summary measures of exposure<br>*Cross-sectional study* - Report numbers of outcome events or summary measures			
Main results	16	(a) Give unadjusted estimates and, if applicable, confounder-adjusted estimates and their precision (e.g., 95% confidence interval). Make clear which confounders were adjusted for and why they were included<br>(b) Report category boundaries when continuous variables were categorized<br>(c) If relevant, consider translating estimates of relative risk into absolute risk for a meaningful time period	Results:<br>clinical outcomes.<br>Table 1, 2, 3.<br>Figure 1, 2, 3.		
Other analyses	17	Report other analyses done—e.g., analyses of subgroups and interactions, and sensitivity analyses	Results:<br>Supplementary Table 1<br>Supplementary Table 2		
**Discussion**					
Key results	18	Summarise key results with reference to study objectives	Discussion:<br>summary of key results provided		
Limitations	19	Discuss limitations of the study, taking into account sources of potential bias or imprecision. Discuss both direction and magnitude of any potential bias	Discussion:<br>extensive description of limitations provided.	RECORD 19.1: Discuss the implications of using data that were not created or collected to answer the specific research question(s). Include discussion of misclassification bias, unmeasured confounding, missing data, and changing eligibility over time, as they pertain to the study being reported.	Discussion:<br>data interpretation in the context of the current literature;<br>extensive description of limitations provided.
Interpretation	20	Give a cautious overall interpretation of results considering objectives,	Discussion:<br>data interpretation in the context of the current literature		

		limitations, multiplicity of analyses, results from similar studies, and other relevant evidence	Discussion: extensive description of study strenghts and limitations provided.		
Generalisability	21	Discuss the generalisability (external validity) of the study results			
**Other Information**					
Funding	22	Give the source of funding and the role of the funders for the present study and, if applicable, for the original study on which the present article is based	Not applicable		
Accessibility of protocol, raw data, and programming code		..	Materials and methods: adherence to TOP guidelines.	RECORD 22.1: Authors should provide information on how to access any supplemental information such as the study protocol, raw data, or programming code.	Materials and methods: adherence to TOP guidelines.

*Reference: Benchimol EI, Smeeth L, Guttmann A, Harron K, Moher D, Petersen I, Sørensen HT, von Elm E, Langan SM, the RECORD Working Committee. The REporting of studies Conducted using Observational Routinely-collected health Data (RECORD) Statement. *PLoS Medicine* 2015; in press.

*Checklist is protected under Creative Commons Attribution (CC BY) license.
